# Sex, Drugs, and Impulse Regulation: A Perspective on Reducing Transmission Risk Behavior and Improving Mental Health Among MSM Living With HIV

**DOI:** 10.3389/fpsyg.2020.01005

**Published:** 2020-05-28

**Authors:** Rachel M. Arends, Thom J. van den Heuvel, Eline G. J. Foeken-Verwoert, Karin J. T. Grintjes, Hans J. G. Keizer, Aart H. Schene, André J. A. M. van der Ven, Arnt F. A. Schellekens

**Affiliations:** ^1^Department of Psychiatry, Radboud University Medical Center, Nijmegen, Netherlands; ^2^Donders Center for Medical Neuroscience, Donders Institute for Brain, Cognition and Behavior, Nijmegen, Netherlands; ^3^Tactus Addiction Care, Deventer, Netherlands; ^4^Scelta, GGNet, Nijmegen, Netherlands; ^5^Youz, Parnassia Group, Arnhem, Netherlands; ^6^Department of Internal Medicine, Radboud University Medical Center, Nijmegen, Netherlands; ^7^Nijmegen Institute for Scientist-Practitioners in Addiction, Nijmegen, Netherlands

**Keywords:** chemsex, HIV, intervention, mental health, MSM, risk behavior

## Abstract

Unprotected sexual contact continues to be a main cause of HIV transmission and poses certain key populations at increased risk for HIV infection. One of the populations at high risk are men who have sex with men. A subset of MSM engages in chemsex, whereby consumption of illicit drugs is used to facilitate or enhance sexual activity. This practice can have several negative consequences, such as sexually transmitted infections (including HIV) and mental health problems (including compulsive sexual behavior, addiction, and mood disorders). In this article, we provide our perspective on the current situation that medical professionals dealing with MSM living with HIV often feel empty-handed in how to deal with these behavioral and psychological issues. Close collaboration between somatic and mental health professionals is key to address treatment needs of people living with HIV, regarding the negative consequences of chemsex and their overall quality of life. In this article, we discuss possibilities for psychological treatment, including behavioral skills training to improve impulse control and reduce compulsive sexual behaviors among MSM living with HIV who persistently engage in sexual transmission risk behavior, based on our experience with implementing such an intervention. Important barriers and facilitators for further implementation of behavioral interventions will be discussed. Reduction of HIV transmission risk behavior is needed to achieve the WHO aim to end HIV as a public health threat by 2030. We propose that close collaboration between somatic and mental health professionals and implementation of behavioral interventions for risk populations are key to achieve this goal.

## Persistent HIV Transmission Risk Behavior Among MSM

HIV remains an important global public health issue (Malekinejad et al., 2008), infecting around 1.8 million people every year (UNAIDS, 2018a). The World Health Organization (WHO) aims to end HIV/AIDS as a public health threat by 2030, as one of the sustainable development goals (WHO, 2016; Bekker et al., 2018). With the increasing availability of combination antiretroviral therapy (cART), HIV infection has transformed into a manageable chronic disease in many parts of the world (Cohen et al., 2013; Deeks et al., 2013). Consequently, the numbers of people living with HIV are increasing, including the physical and emotional challenges of living with HIV (Crepaz et al., 2008; Mutumba and Harper, 2015).

Around 57% of all new HIV infections in Western societies occur among men who have sex with men (MSM), making them a major risk population (UNAIDS, 2018b). MSM have a 28 times higher risk of contracting HIV, compared to the general population (UNAIDS, 2018b). This is mainly driven by sexual transmission risk behavior, sometimes combined with substance use, in a subpopulation of MSM (Benotsch et al., 1999; Purcell et al., 2001; Pufall et al., 2018). While cART has significantly reduced HIV transmission, access to cART also caused an increase in unsafe sexual practices since the mid-90s. This contributed to an annually increasing rate of sexually transmitted infections (STIs) among MSM, including syphilis, gonorrhea, hepatitis, and chlamydia (Workowski and Bolan, 2015; UNAIDS, 2016). This development also stirred antibiotic resistance in some of these infections and is accompanied by high costs for treatment of e.g., hepatitis C (Nelson, 2003; Crowther-Gibson et al., 2011; Unemo and Shafer, 2011).

Various studies show that around 40% of MSM living with HIV report persisting sexual transmission risk behavior (Benotsch et al., 1999; Hospers et al., 2005; Van Kesteren et al., 2007; Crepaz et al., 2009), with recent unprotected sexual practices being reported by 39–66% of seropositive MSM (Benotsch et al., 1999). Furthermore, there is a growing trend for MSM to participate in group sex under the influence of psychoactive drugs, most commonly known as “chemsex” or “party and play” (Evers et al., 2020). Frequently used drugs to increase sexual experiences include crystal methamphetamine (when it is injected called “slamming”), mephedrone and gamma-hydroxybutyrate (GHB) (Stuart, 2013; Moncrieff and Friend, 2014; McCall et al., 2015; Schmidt et al., 2016; González-Baeza et al., 2018; Pufall et al., 2018). Chemsex increases the risk of transmission of HIV and other STIs (Purcell et al., 2001; Hegazi et al., 2017; Sewell et al., 2017; Pufall et al., 2018). Recent studies from the UK and the Netherlands showed that around 30–40% of MSM report to have had sex in the context of substance use (Pufall et al., 2018; Evers et al., 2020). Evers et al. (2020) also observed that of the MSM engaging in chemsex, 23% reported a need for professional counseling, mainly MSM who engaged in chemsex more often, and the most reported subject to discuss was increasing self-control (reported by 52%).

In about 14–22% of MSM living with HIV, ongoing transmission risk behavior develops into compulsive sexual behavior (CSB) (Benotsch et al., 1999; Parsons et al., 2017; Brown et al., 2018). CSB is characterized by a preoccupation with one’s sexual desires and an inability to control them, often resulting in more frequent and unsafe sexual encounters (Benotsch et al., 1999; Dew and Chaney, 2005). For people with CSB, having sex can become an alternative strategy to cope with negative emotions such as loneliness, anxiety, stress (Collins et al., 2006; Torres and Gore-Felton, 2007), low self-esteem (Benotsch et al., 1999; Dew and Chaney, 2005), or traumatic stress (Goodman and Fallot, 1998; Stevens et al., 2003). Among MSM living with HIV, CSB is of particular relevance, since it can further contribute to the transmission of STIs, including HIV (Benotsch et al., 1999).

Finally, other mental health issues have also been associated with persisting transmission risk behavior among MSM living with HIV (Crepaz and Marks, 2002; Prince et al., 2007; Bonar et al., 2018). Approximately half of the people living with HIV are diagnosed with a psychiatric disorder during their lives (Crepaz et al., 2008). Most common psychiatric conditions among MSM living with HIV are depression (14–48% compared with 6–37% in the general population), anxiety disorders (16–40% compared to 6–18%), and substance use disorders (7–65% compared to 4–11%) (Dew et al., 1997; ESEMeD/MHEDEA, 2004; Baumeister and Härter, 2007; Crepaz et al., 2008; Myer et al., 2008). Mental health issues contribute to a lower quality of life (Sherbourne et al., 2000; Berg et al., 2004), and decreased adherence to cART in this population (Pence et al., 2007). Where mental health services often lack expertise regarding HIV, cART, and HIV-specific mental health aspects, somatic healthcare professionals often feel empty-handed when it comes to mental health and addiction issues in their patients (Soto et al., 2004; Cournos et al., 2005; Moncrieff and Friend, 2014; McCall et al., 2015).

In this paper, we provide our perspective on a closer collaboration between somatic and behavioral health professionals, which could enhance the quality of care, improve the mental health and self-control of patients, and decrease societal public health issues regarding this population (Soto et al., 2004; Cargill and Stone, 2005; Moncrieff and Friend, 2014).

## Behavioral Intervention: Ideas and Experiences

Various studies stress the importance of behavioral interventions in order to reduce transmission risk behavior among MSM (Johnson et al., 2008; Berg, 2009; Higa et al., 2013; Krishnaratne et al., 2016; Brown et al., 2019). Studies show that behavioral interventions that focus on emotion and impulse regulation can increase quality of life, and reduce transmission risk behavior among key populations, including MSM living with HIV (Johnson et al., 2008; Farmer and Golden, 2009; Neacsiu et al., 2014). In addition, previous studies have shown associations between impulsiveness and transmission risk behavior in various key-populations (Horvath and Zuckerman, 1993; Semple et al., 2006; Jemmott III, Jemmott et al., 2015; Danko et al., 2016; Arends et al., 2019). Therefore, we postulate that interventions aiming to reduce transmission risk behavior should also target elements as impulse regulation skills. Group therapy might be a preferable option for such interventions, as it helps individuals feel less isolated and ashamed (Schreiber et al., 2011). However, current evidence is also limited, mainly due to methodological limitations, including small sample sizes and observational designs (Johnson et al., 2008). Furthermore, there is a lack of scientifically tested treatment behavioral intervention protocols for chemsex and sexual compulsivity for MSM living with HIV (Evers et al., 2020; Stevens et al., 2020).

To explore the possibilities for future studies and implementation of a behavioral intervention protocol for MSM living with HIV who show persistent sexual transmission risk behavior, we developed a behavioral intervention combining different treatment elements (based on cognitive and dialectical behavior therapy) previously shown to be effective in improving self-awareness, emotion and impulse regulation, and reducing risky behaviors such as substance use, self-harm or sexual risk behavior (Wagner et al., 2004; Crepaz et al., 2008; Covey et al., 2016; Cristea et al., 2017; Maffei et al., 2018; DeCou et al., 2019; Ben-Porath et al., 2020). To the best of our knowledge, this is the first time a cognitive-dialectical behavior therapy protocol is used, mainly focusing on developing insight in one’s dysfunctional/risky behavior and increasing coping skills (related to reduced self-management and lowered impulse and emotion regulation) (Neacsiu et al., 2014) in order to reduce sexual transmission risk behavior among MSM living with HIV.

The exploratory intervention consisted of 10 weekly sessions of 2.5 h group therapy, guided by two psychologists. The intervention consisted of self-acceptance exercises and practicing self-control skills, and discussing people’s diary notes and homework assignments. It contained elements of motivational interviewing, mindfulness-based therapy, self-regulation, and risk management skills training (see Table 1 for a general outline of the intervention program). The intervention aimed to assist patients with increasing insight in their sexual- and substance-related behavior (incl. related problems) and providing ways to learn alternative strategies for coping with problems and negative emotions, instead of using drugs or having sex.

**TABLE 1:** Overview of treatment components related to therapeutic factors.

Session	Subject
1	Introduction, psycho-education, and start with general skills training
2	Mindfulness, creating awareness of current problems
3	Mindfulness and pros/cons of current behavior
4	Distress tolerance, dealing with mental health issues
5	Distress tolerance, alternative ways of coping with negative affect I/O sex or substance use
6	Emotion regulation and impulse control
7	Emotion regulation and self-esteem
8	Interpersonal effectiveness, set boundaries
9	Interpersonal effectiveness, maintaining and severing relationships
10	Repeating lessons learned, training evaluation and making aftercare appointments

All MSM living with HIV being treated at our referral hospital in the eastern part of The Netherlands (*n* ≈ 425), who had multiple STIs in previous year (*n* = 70), and did not have any acute somatic or mental health issues (exclusion criteria), were offered the possibility to participate in the behavioral intervention by their treating nurse practitioners. Subsequently, men who expressed a wish to change their substance- and/or sexual-related risk behavior were approached and screened for participation by a psychologist. Finally, 12 MSM living with HIV participated in the intervention. On average they were 42 years old (range: 25–54). All participating men reported symptoms of CSB and substance use in the previous month. Furthermore, they reported clinically significant levels of anxiety (41%), stress (32%), and/or depression (38%). In a qualitative evaluation, they all reported increased awareness of their transmission risk behavior and its consequences, and more than half (55%) also experienced behavioral change after the behavioral intervention. Most participants (82%) reported that the training provided them with new insights into their sexual behavior related problems. Furthermore, all reported having learned alternative strategies to cope with problems and negative emotions, instead of using drugs or having sex. Finally, more than half of the participants (55%) reported the impulse and emotion regulation skills to be most helpful for them.

Other relevant themes identified during the intervention were lack of self-confidence, problems in intimate relationships, exposure to traumatic experiences, drug abuse, and a lower perceived quality of life. These topics may be of interest for future studies exploring the effects of a behavioral intervention in this target population. Future studies should thereby not only focus on reducing sexual transmission risk behavior and mental health problems but also include positive outcome measures of personal recovery, well-being and (post-traumatic) growth (Kamen et al., 2016; Willie et al., 2016; Moitra et al., 2020). The main barriers that were encountered during recruitment were related to fear for change, feelings of shame or guilt about one’s behavior (Gilliland et al., 2011), stigma and concerns about privacy. In addition, some patients expressed little self-efficacy or insight into their behavior. Previous studies have shown that this impedes help-seeking behavior, behavioral change, and adoption of a healthier lifestyle (Gilliland et al., 2011; Schwarzer, 2014).

## Upscaling Targeted Interventions to Reduce Transmission Risk Behavior

People who live with HIV often know the risks of transmission risk behavior, but some continue to engage in risky practices despite known negative consequences (McKirnan et al., 1996; Montano and Kasprzyk, 2015). Thus, only providing knowledge on transmission risk behavior and its potential consequences is unlikely to result in sufficient behavioral change (McKirnan et al., 1996; Smith et al., 2012). Based on our exploratory experience with a behavioral group intervention, and supported by several previous studies, we postulate that behavioral intervention is key to achieve WHO sustainable development goals on HIV. Behavioral interventions can contribute to increase insight in one’s behavior and facilitate behavioral change among people living with HIV (Herbst et al., 2005; Johnson et al., 2008; Covey et al., 2016). Interventions, such as reported here, can improve impulse and emotion regulation skills and therefore reduce “self-medication” with sex and/or substance use (as part of transmission risk behavior) and advance mental health among people living with HIV (Margolin et al., 2007; Salomon et al., 2009; Arends et al., 2019).

This approach requires interdisciplinary collaboration, in order to address unmet mental health needs in people living with HIV. Through interdisciplinary collaboration HIV healthcare professionals can offer more comprehensive assessment and treatment, and mental health care workers can integrate specific HIV-related aspects into their care routines (Sherer et al., 2002; Soto et al., 2004; Cargill and Stone, 2005; Halkitis et al., 2013; Moncrieff and Friend, 2014; UNFPA, 2015). For instance, mental healthcare workers previously reported a need for improved HIV-related knowledge to expand services for people living with HIV and addiction problems (Montague et al., 2015).

Behavioral interventions targeting transmission risks in specific key populations as described here, could be part of a “micro-elimination” approach to achieve the HIV/AIDS-related WHO sustainable development goals. Micro-elimination concerns the reduction of infections to zero in specific key populations (Lazarus et al., 2018). To achieve this, a people-centered organization of health care is required, where a specific population is offered suitable physical and mental healthcare (Mahajan et al., 2008; Lazarus et al., 2017). Though MSM with mental health issues and persistent transmission risk behavior constitute a relatively small proportion of all people living with HIV, the high likelihood of HIV transmission within this population makes them a priority target for preventive interventions (Bourne et al., 2015).

A major issue regarding psychological support is the motivation for treatment. There are various reasons why MSM living with HIV with persisting transmission risk behavior might not ask for help or not participate in a behavioral intervention. Patients often only seek help for their somatic problems, probably due to stigma and secrecy that surround same-sex behavior, sex-related problems, mental health issues or substance use (McKirnan et al., 1996; McCall et al., 2015; Gama et al., 2017). Previous studies show that issues regarding participating in a behavioral intervention can include anxiety to talk about risk behavior, perceived stigma, feelings of shame, and privacy issues as major barriers to participating in the behavioral skills training (Gilliland et al., 2011; Kamarulzaman and Altice, 2015; Raifman et al., 2017). Certain studies suggest that online approaches (eHealth) can circumvent some of these barriers (Amichai-Hamburger et al., 2014; Connaughton and McCabe, 2017) and can be effective in reducing transmission risk behaviors among key populations (Johnson et al., 2008). These kinds of eHealth approaches might be able to reach patients in need who would otherwise not ask for help (Postel et al., 2008). Future studies should investigate whether the transformation of face-to-face interventions, as described here, into eHealth applications holds promise for reducing HIV transmission (e.g., related to CSB or chemsex) in relevant key-populations. Furthermore, future studies should apply a (quasi) experimental study designs including a control group, in order to examine the effectiveness of such an intervention in reducing sexual transmission risk behavior among MSM living with HIV, and improving personal well-being.

Taken together, persisting transmission risk behavior among MSM living with HIV can have a major impact on their quality of life and is of utmost importance from a public health perspective. Interdisciplinary collaboration to develop and implement a tailored behavioral intervention might help to reduce transmission risk behavior and improve quality of life in these patients (Cournos et al., 2005), and might save costs by preventing transmission of STI’s (Herbst et al., 2005; Lin et al., 2016). Future studies should provide further evidence for efficacy, and explore ways to tackle barriers for participation in such interventions, for instance by providing online treatment or integrating somatic and mental health care services. We strongly believe that these kinds of behavioral approaches are needed to reach the WHO sustainable development goal to end the HIV/AIDS epidemic by 2030.

## Data Availability Statement

The datasets generated for this study are available on request to the corresponding author.

## Ethics Statement

The study that was mentioned in this manuscript involved human participants and was reviewed and approved by the Central Committee on Research Involving Human Subjects region Arnhem-Nijmegen. Furthermore, the participants provided their written informed consent to take part in the study.

## Author Contributions

RA wrote the main parts of the manuscript. TH, EF-V, KG, AFS, AHS, and AV gave relevant feedback on the content. AFS provided major feedback and adjustments of text elements. All authors read and approved the final manuscript.

## Conflict of Interest

RA was employed by Tactus Addiction Care, TH was employed by Scelta, and EF-V was employed by Youz. The remaining authors declare that the research was conducted in the absence of any commercial or financial relationships that could be construed as a potential conflict of interest.

## Abbreviations

AIDS: acquired immunodeficiency disease
cART: combination antiretroviral treatment
CSB: compulsive sexual behavior
GHB: gamma-hydroxybutyrate
HIV: human immunodeficiency virus
MSM: men who have sex with men
STI(s): sexually transmitted infection(s)
WHO: World Health Organization.
